# Sulfur and Peroxide Vulcanization of the Blends Based on Styrene–Butadiene Rubber, Ethylene–Propylene–Diene Monomer Rubber and Their Combinations

**DOI:** 10.3390/ma17112718

**Published:** 2024-06-03

**Authors:** Ján Kruželák, Andrea Kvasničáková, Michaela Džuganová, Jan Hanzlik, Martin Bednarik, Ivan Chodák, Ivan Hudec

**Affiliations:** 1 Department of Plastics, Rubber and Fibres, Faculty of Chemical and Food Technology, Slovak University of Technology in Bratislava, Radlinského 9, 812 37 Bratislava, Slovakia; andrea.kvasnicakova@stuba.sk (A.K.); michaela.dzuganova@stuba.sk (M.D.); ivan.hudec@stuba.sk (I.H.); 2 Faculty of Technology, Tomas Bata University in Zlin, Vavreckova 5669, 760 01 Zlin, Czech Republic; j_hanzlik@utb.cz (J.H.); mbednarik@utb.cz (M.B.); 3 Polymer Institute, Slovak Academy of Sciences, Dúbravská cesta 9, 845 41 Bratislava, Slovakia; ivan.chodak@savba.sk

**Keywords:** rubber blends, sulfur curing systems, peroxide curing systems, cross-linking, properties

## Abstract

Rubber blends based on styrene–butadiene rubber, ethylene–propylene–diene monomer rubber and a combination of both rubbers were cured with different sulfur and peroxide curing systems. In sulfur curing systems, two type of accelerators, namely tetramethylthiuram disulfide, N-cyclohexyl-2-benzothiazole sulfenamide, and combinations of both accelerators were used. In peroxide curing systems, dicumyl peroxide, and a combination of dicumyl peroxide with zinc diacrylate or zinc dimethacrylate, respectively, were applied. The work was aimed at investigating the effect of curing systems composition as well as the type of rubber or rubber combinations on the curing process, cross-link density and physical–mechanical properties of vulcanizates. The dynamic mechanical properties of the selected vulcanizates were examined too. The results revealed a correlation between the cross-link density and physical–mechanical properties. Similarly, there was a certain correlation between the cross-linking degree and glass transition temperature. The tensile strength of vulcanizates based on rubber combinations was higher when compared to that based on pure rubbers, which points out the fact that in rubber combinations, not only are the features of both elastomers combined, but improvement in the tensile characteristics can also be achieved. When compared to vulcanizates cured with dicumyl peroxide, materials cured with a sulfur system exhibited higher tensile strength. With the application of co-agents in peroxide vulcanization, the tensile strength overcame the tensile behavior of sulfur-cured vulcanizates.

## 1. Introduction

Styrene–butadiene rubber (SBR) is general-purpose rubber with an unsaturated polymer backbone. It is the most widely used rubber in rubber technologies, applied in the production of tires, conveyor belts, hoses, rubber carpeting, shoe soles and in other applications. The properties of SBR are highly dependent on the proportion ratio between butadiene and styrene units. Increasing the amount of styrene leads to higher tensile and tear strength, higher hardness, higher abrasion resistance and higher processability. On the other hand, the elasticity decreases with an increase in styrene content. Due to a high degree of unsaturation, SBR exhibits limited resistance to degradation factors, like oxygen and ozone, and low resistance to solvents and chemicals [1,2].

Ethylene–propylene–diene monomer rubber EPDM is one of the most versatile specialty type rubbers, with excellent electro-insulating properties, which are retained in high-humidity environments and increased temperatures [3]. It also exhibits very good elastic properties at low temperatures, a low compression set and good resistance against abrasion and fracturing. The double bonds are situated only in side groups of non-conjugated diene, meaning that the main polymer chain is saturated. EPDM thus demonstrates good resistance to thermo-oxidative and ozone ageing [4]. It has also good resistance to polar solvents, water, and steam. EPDM is widely used for manufacturing of conveyor belts, hydraulic and industrial hoses, sealants, cable sheeting, connectors, roof membranes, asphalt modifications, etc. As the concentration of double bonds in EPDM is low, this rubber has a low propensity to vulcanization with sulfur curing systems.

As each rubber has some advantages as well as drawbacks, the main aim of rubber combinations is to provide the benefits of one rubber or to suppress the disadvantages of another one in their mutual combination. The combination of SBR with EPDM leads to higher resistance of the compounds against thermo-oxidative and ozone ageing and to chemical attacks. Abrasion resistance of the compounds is also improved.

Vulcanization, often referred to as curing is a very important process in rubber technology leading to the formation of a three-dimensional cross-linked network structure within the rubber matrix. This network increases physical–mechanical properties and elasticity, while reducing hysteresis and plasticity. Different vulcanization systems can be used for cross-linking rubber matrices. The selection of a curing system usually depends on the type of rubber or rubbers in the compound and on the properties of the final materials too.

Sulfur vulcanization is the most frequently used method for cross-linking rubber compounds, accounting around 90% of products. It is very complex process, generally running in several stages. Sulfur vulcanization systems generally consist of activators, accelerators, and sulfur. Activators and accelerators are very important parts of sulfur vulcanization systems; they increase reaction speed, decrease the vulcanization temperature, and increase the efficiency of sulfur to form cross-links between rubber chain segments. The most common activators for sulfur vulcanization are zinc oxide in combination with fatty acid, like stearic acid or palmitic acid. A lot of accelerators have been used in sulfur curing systems; they are organic substances, mostly containing sulfur and nitrogen in their structures. They differ in their chemical structure and activity in the vulcanization process. During sulfur vulcanization, activators together with accelerators form a salt that reacts with sulfur to generate an active sulfurating agent. A primary vulcanizing network with dominant polysulfidic cross-links is formed in the second stage. The restructuring of polysulfidic cross-links into di- and monosulfic cross-links and the modifications of rubber chains occur in the final stage, which leads to the generation of a final three-dimensional cross-linked network [5,6,7]. Through the sulfur curing process, rubber chain segments are cross-linked with sulfidic cross-links with a different number of sulfur atoms in sulfur bridges (namely mono-, di- and polysulfidic cross-links) [8,9].

Organic peroxides are mainly used for cross-linking compounds based on saturated elastomers, which cannot be cured with sulfur. Although, they are also effective for cross-linking of unsaturated rubbers. During the peroxide curing process, organic peroxide first undergoes homolytic dissociation by the cleavage of an oxygen–oxygen bond [10,11]. This leads to the formation of primary peroxide radicals, which can be decomposed into secondary radical fragments. Both types of radicals are active in vulcanization process. Peroxide radicals can react with rubber chains by adding to the double bonds or by hydrogen abstraction. Both reaction mechanisms lead to the formation of rubber radicals, which undergo recombination to form carbon–carbon bonds between the chain segments [12,13,14]. C-C cross-links have higher bonding energy when compared to sulfidic cross-links (C—S_x_—C < 252 kJ·mol^−1^; C—S_2_—C < 268 kJ·mol^−1^; C—S—C < 285 kJ·mol^−1^; C—C < 352 kJ·mol^−1^), and thus the main feature of peroxide-cured vulcanizates are good heat ageing stability, good resistance to thermo-oxidative ageing, and a low compression set [15,16]. However, when compared to sulfur-cured vulcanizates, they have worse physical–mechanical properties, like poorer tensile and tear strength and worse dynamic properties [17].

To increase the cross-linking efficiency of rubber compounds with organic peroxides, low molecular organic compounds with activated double bonds, the so-called co-agents, are often applied [18,19]. Co-agents can boost the peroxide vulcanization process by suppressing side reactions like chain scission or disproportionation. But the main reason for their positive effect on the curing process is that they actively participate in the formation of extra cross-links. This subsequently leads not only to the increase in cross-link density, but the structure and the quality of the formed cross-links is changed too. This contributes to improvements in the physical–mechanical properties of peroxide-cured vulcanizates [20,21,22,23].

Both SBR and EPDM can be cured with peroxide as well as sulfur curing systems. The reaction mechanisms of sulfur and peroxide curing of both rubbers have been described in several scientific studies, for example, refs. [24,25,26,27]. The current study deals with the investigation of several sulfur and peroxide curing systems on pure SBR and EPDM rubbers, as well as their mutual combinations. This study investigates the influence of curing system compositions on the vulcanization process, cross-link density, physical–mechanical, and dynamic–mechanical properties. The relation between the cross-link density and the structure of the formed cross-links in relation to the tested properties is outlined.

Peroxide curing systems have never been reported to have been applied for cross-linking of rubber blends based on SBR and EPDM combinations. Thus, this work is focused on comparing the cross-linking process of peroxide- and sulfur-based curing systems of SBR and EPDM blends in relation to their physical– and dynamical–mechanical properties.

## 2. Experimental

### 2.1. Materials

Styrene–butadiene rubber (SBR, Kralex 1502, styrene content—23.5 wt.%) prepared by cold emulsion polymerization was supplied from Synthos Kralupy, a.s. Kralupy nad Vltavou, Czech Republic. Ethylene–propylene–diene monomer rubber (EPDM, type KEP 570F, ethylene content—70 wt.%, non-conjugated 5-ethylidene-2-norbornene monomer (ENB) content—4.5 wt.%) was provided by Kumho Polychem Co., Ltd., Seoul, Republic of Korea. Sulfur curing system consisted of activators (stearic acid and zinc oxide), accelerators (N-cyclohexyl-2-benzothiazole sulfenamide (CBS), tetramethylthiuram disulfide (TMTD)) and sulfur. The chemicals for the sulfur curing system were provided by Vegum a.s., Dolné Vestenice, Slovak Republic). Dicumyl peroxide (DCP) with 99% purity was used as a peroxide curing agent. Zinc diacrylate (ZDA) and dimethacrylate (ZDMA) were used as co-agents in peroxide vulcanization. The chemicals for the peroxide curing system were supplied from Sigma-Aldrich, Burlington, MA, USA.

### 2.2. Methods

#### 2.2.1. Preparation and Curing of Rubber Compounds

Five types of rubber formulations based on SBR and EPDM were fabricated. The first rubber blend was based on SBR, while the last was based on EPDM. In the other three types of rubber formulations, the mutual ratio of both rubbers was changed. The cross-linking of rubber blends was performed using sulfur or peroxide curing systems. The amount of activators and sulfur was kept constant in all rubber formulations. The rubber compositions differed depending on the type of accelerator. In the first series of rubber blends, N-cyclohexyl-2-benzothiazole sulfenamide was used in an amount 1.5 phr. Similarly, in the second series, tetramethylthiuram disulfide was applied in the same amount, 1.5 phr. In the third series, a combination of both accelerators in a ratio of 1 phr to 1 phr was used. The composition of rubber blends with the sulfur curing system is summarized in Table 1 and Table 2.

In the first series of rubber blends cured with peroxide systems, dicumyl peroxide was exclusively used for cross-linking the rubber blends (Table 3). In next two series, dicumyl peroxide in combination with zinc diacrylate or zinc dimethacrylate, respectively, was used (Table 4).

The fabrication of rubber blends was performed in a laboratory kneading machine Brabender (Brabender GmbH & Co. KG, Duisburg, Germany) in two mixing steps. The overall mixing time was 11 min at a temperature of 90 °C at 50 rpm. The rubber or rubbers were first put into the chamber and were plasticated for 2.5 min. Then, zinc oxide and stearic acid were added, and the rubber blends were mixed for 4.5 min at 90 °C and 50 rpm. The rubber blends were taken out from the chamber, cooled down, and homogenized using a two-roll mill. In the second step, sulfur and the accelerator or a combination of accelerators were introduced, and the rubber systems were compounded for 4 min at 90 °C. After mixing, the rubber compounds were sheeted in a two-roll mill.

The compounding process of the rubber formulations with a peroxide curing system was very similar. In the first step, only the rubber or rubbers in their mutual combinations were mixed and plasticated. In the second step, DCP or a combination of DCP with a co-agent (ZDA or ZDMA) was applied, and the rubber blends were mixed for 4 min at 90 °C and 50 rpm. The final step was homogenization and sheeting of the compounds using the two-roll mill.

The vulcanization process was performed at 160 °C and a pressure of 15 MPa in a hydraulic press Fontijne (Fontijne, Vlaardingen, The Netherlands). The time of vulcanization was identical with the optimum cure time of each rubber blend, which was determined from corresponding curing isotherms. After curing, thin sheets with dimensions 15 × 15 cm and thickness 2 mm were obtained.

#### 2.2.2. Determination of Curing Characteristics

Curing characteristics of the blends were determined from corresponding curing isotherms, which were investigated using an oscillatory rheometer MDR 2000 (Alpha Technologies, Akron, OH, USA).

The investigated curing parameters were as follows:

∆*M* (dN·m)—torque difference, the difference between the maximum and minimum torque (∆*M* = *M_H_* − *M_L_*).

*t_c_*_90_ (min)—optimum curing time.

*t_s_*_1_ (min)—scorch time.

*R* (dN·m·min^−1^)—curing rate, defined as:

$$R=\frac{M_{c90}-M_{s1}}{t_{c90}-t_{s1}}$$

*M_c_*_90_—torque at *t_c_*_90_.

*M_s_*_1_—torque at *t_s_*_1_.

#### 2.2.3. Determination of Cross-Link Density

The cross-link density *ν* was determined based on the equilibrium swelling of vulcanizates in xylene. The weighted dried samples were placed into xylene in which they swelled within time. The weight of samples was measured every hour until equilibrium swelling was reached. During the measurement, the solvent diffused into the rubber and disrupted almost all physical interactions within the rubber matrix. The result was the determination of chemical cross-link density, i.e., the concentration of chemical cross-links within the rubber compounds. The experiments were carried out at a laboratory temperature, and the swelling time was equal to 30 h. The Flory–Rehner equation [28] was then used to calculate the cross-link density based upon the equilibrium swelling state.

#### 2.2.4. Investigation of Physical–Mechanical Characteristics

Zwick Roell/Z 2.5 appliance (Zwick GmbH & Co. KG, Ulm, Germany) was used to evaluate tensile properties of vulcanizates. The tests were performed in accordance with the valid international technical standards (ISO 37) and the cross-head speed of the measuring device was set up to 500 mm.min^−1^. Dumbbell-shaped test samples (width 6.4 mm, length 80 mm, thickness 2 mm) were used for measurements. The hardness was measured by using durometer and was expressed in Shore A.

#### 2.2.5. Determination of Dynamical–Mechanical Properties

The dynamical–mechanical performances of vulcanizates were obtained using a dynamical-mechanical analyzer DMTA MkIII, fy Rheometric Scientific (Piscataway, NJ, USA), following the international standard ISO 6721-11:2019. The samples were analyzed in tensile mode at a frequency of 10 Hz, an amplitude of dynamic deformation of 64 μm, and a static force of 0.2 N in a temperature range of −60 °C to 80 °C. The heating rate was 2 °C per min.

## 3. Results and Discussion

### 3.1. Vulcanization Process and Physical–Mechanical Properties of Sulfur-Cured Rubber Compounds

**Figure 1:**
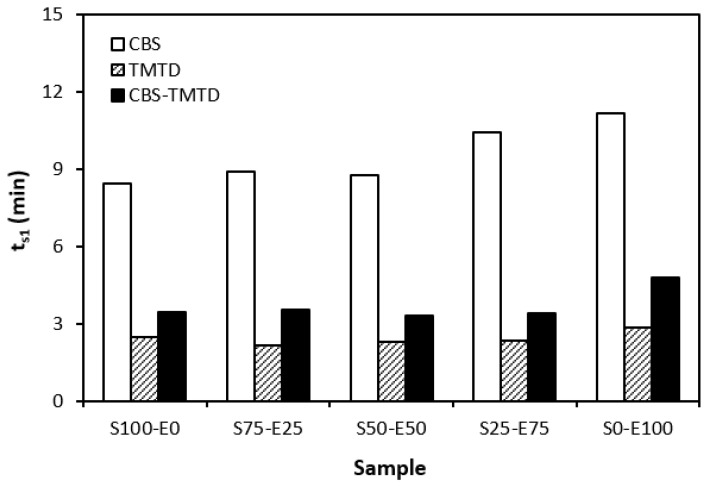
Scorch time *t_s_*_1_ rubber blends cured with sulfur systems.

**Figure 2:**
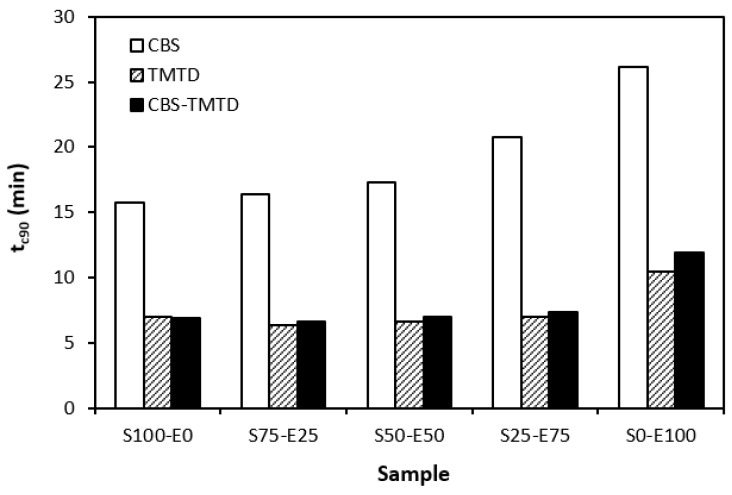
Optimum cure time *t_c_*_90_ of rubber blends cured with sulfur systems.

The influence of curing system compositions on the vulcanization characteristics of rubber formulations was examined through the determination of scorch time *t_s_*_1_, optimum cure time *t_c_*_90_, curing rate *R*, and the difference between the maximum and minimum torque ∆*M.* As seen in Figure 1, the highest scorch time was that of rubber blends cured in the presence of a CBS accelerator. On the other hand, the lowest *t_s_*_1_ was that of the rubber formulations cured with TMTD. It can be stated that the scorch time showed a slight increasing trend with an increasing ratio of EPDM in rubber combinations, and the highest scorch time was that of rubber formulations based on EPDM (S0–E100). Looking at Figure 2, one can see very similar dependences of optimum cure time on the used accelerators. The highest optimum cure time was that of rubber blends cured in the presence of CBS. From Figure 2, it also becomes apparent that the optimum cure time of CBS-based formulations increased with an increasing ratio of EPDM. When compared to rubber blends based on SBR (S100–E0), the *t_c_*_90_ of the blend based on EPDM (S0–E100) was prolonged by more than 10 min (from 16 min to more than 26 min). The rubber blends cured in the presence of TMTD or the combination CBS-TMTD required an almost identical amount of time for their optimum cross-linking. Again, the highest *t_c_*_90_ was exhibited by the S0–E100 blends.

The dependencies of the optimum cure time as well as the scorch time on the type of accelerator used relate with the character and structure of the accelerators. CBS belongs to the class of fast accelerators of the sulfenamide type that are characterized by a long induction period (delayed action accelerators). Thus, the blends cured with CBS exhibited the longest scorch time. TMTD is from a group of very fast accelerators, which indicates that the curing process of rubber formulations proceeds faster. This corresponds with a shorter scorch time and optimum cure time of the corresponding blends. When combining both CBS and TMTD, the optimum cure time was not changed, while slight prolongation of the scorch time was recorded in comparison with the equivalent TMTD-cured rubber blends. Although the prolongation of scorch time was not very significant, it is a positive aspect regarding the safe processability of rubber blends into final cross-linked materials. The scorch time or induction period represents the time during which the cross-linking does not occur yet. The mutual interactions among the additives of the curing system occur, and the materials must be heated uniformly in its entire volume. This is a very important factor for achieving homogenous distribution of the formed cross-links within the rubber matrix and thus forming a spatially uniform, three-dimensional cross-linked structure. When this is achieved, the following vulcanization can proceed quickly, resulting in time and economic benefits. The short optimum vulcanization time of rubber blends with a combination of accelerators (CBS-TMTD) can be attributed to the presence of a very fast accelerator (TMTD) on one hand, and to the increase in the overall accelerator content (2 phr) on the other hand. In general, the more activity there is and the higher the amount of accelerators, the faster the vulcanization process [13].

It was recorded that the curing process of the materials based on EPDM was longer than that of the corresponding blends based on SBR. Considering that SBR is highly unsaturated rubber, the concentration of double bonds in its structure is much higher when compared to EPDM (the double bonds are situated only in non-conjugated ENB monomer units, the concentration of which is only 4.5 wt.% in EPDM). Overall, the lower the concentration of the double bonds in the rubber structure, the slower the vulcanization process.

**Figure 3:**
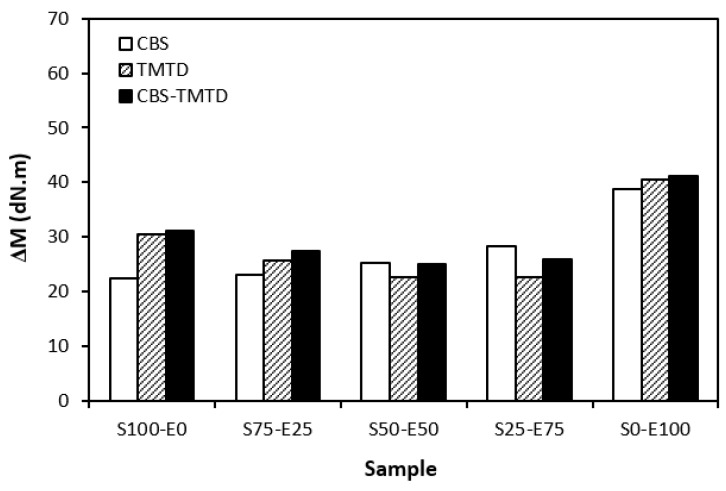
The difference between the maximum and minimum torque Δ*M* of rubber blends cured with sulfur systems.

**Figure 4:**
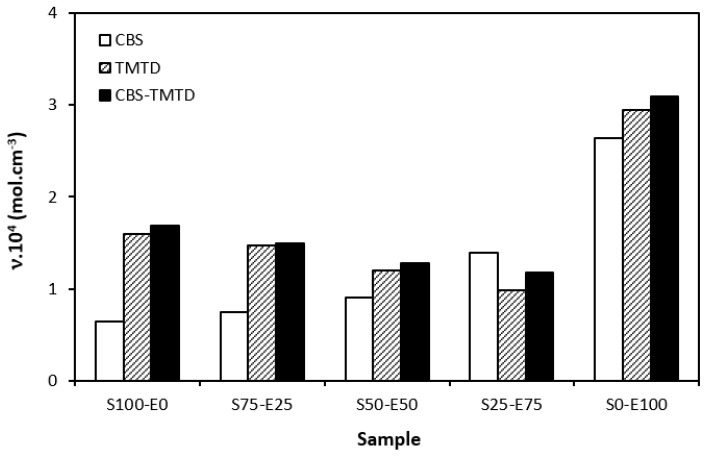
Cross-link density *ν* of vulcanizates cured with sulfur systems.

The difference between the maximum and minimum torque usually relates with the number of cross-links formed within the rubber matrix and expresses the degree of transformation of uncured rubber compound into vulcanizate. Generally speaking, the higher the torque difference, the higher the cross-link density. However, this is valid mainly for unfilled rubber compounds. As seen in Figure 3, the biggest difference between the maximum and minimum torque was exhibited by rubber blends based on EPDM (S0–E100). The differences in the Δ*M* values of the dependence on the type of accelerator or accelerators combinations were minimal. Looking at Figure 4, one can see a certain correlation between cross-link density and torque difference. This means that the highest torque difference in the blends designated S0–E100 was reflected in their highest cross-linking degree. It is interesting that the cross-link density of the materials with CBS showed an increasing trend with an increasing ratio of EPDM in rubber combinations. On the other hand, the cross-link density of vulcanizates cured in the presence of TMTD and the combination CBS-TMTD tended to decrease with an increasing ratio of EPDM up to the composition S25–E75. Then, a significant increase in the degree of cross-linking occurred for the vulcanizate S0–E100. When comparing the curing systems, the highest cross-link density was exhibited by rubber blends cured with the CBS-TMTD combination. TMTD acts not only as accelerator, but also as a sulfur donor due to sulfur bridges in its structure and, thus, it contributes to the cross-links formation. The highest cross-link density of vulcanizates based on EPDM seems to be surprising, as EPDM has a low value of unsaturation and, therefore, the cross-link density was expected to be lower when compared to vulcanizates based on highly unsaturated SBR. A possible explanation could be that the EPDM might have a highly branched structure and the physical entanglements linked with chemical linkages can act as additional cross-links.

**Figure 5:**
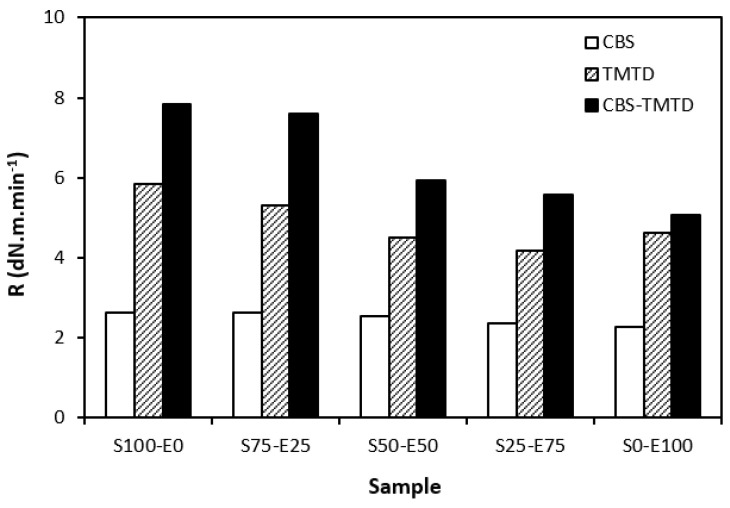
Curing rate *R* of rubber blends cured with sulfur systems.

From Figure 5, it becomes apparent that the lowest curing rate was exhibited by formulations cured with CBS with almost no dependence on the type of rubber or their combinations. The application of TMTD resulted in a higher curing rate, which was found to slightly decrease with an increasing ratio of EPDM in rubber combinations. The highest curing rate was demonstrated by the materials cured with the CBS-TMTD combination with the highest influence on the type of rubber or rubber combinations. The cure rate accounts for not only the differences between the optimum cure time and scorch time, but also the differences between the torque at the optimum cure time and scorch time; thus, it demonstrates the change process of an uncured blend into a vulcanizate. The highest curing rate was that of the blends based on SBR (S100–E0), which suggests that the curing process of equivalent rubber blends proceeded the fastest.

**Figure 6:**
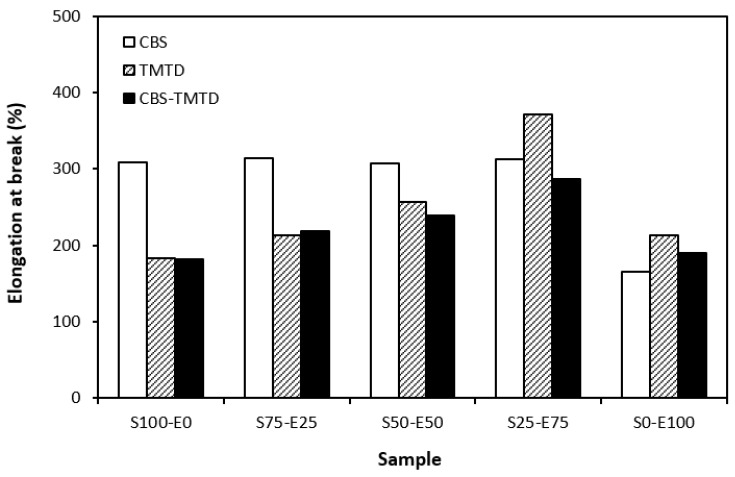
Elongation at break of vulcanizates cured with sulfur systems.

**Figure 7:**
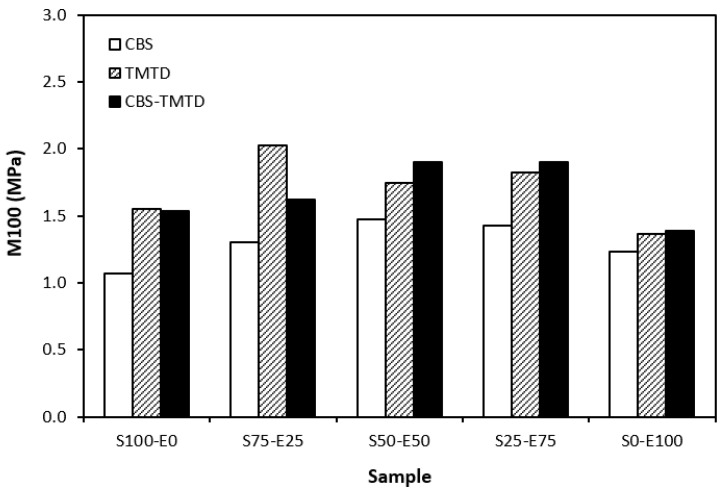
Modulus M100 of vulcanizates cured with sulfur systems.

**Figure 8:**
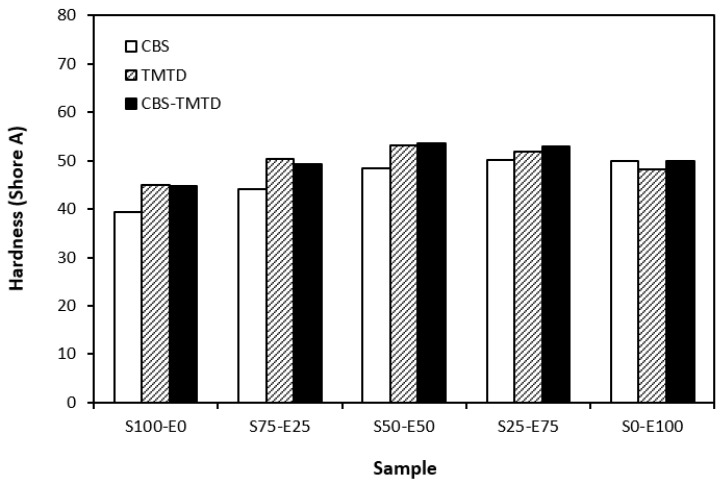
Hardness of vulcanizates cured with sulfur systems.

**Figure 9:**
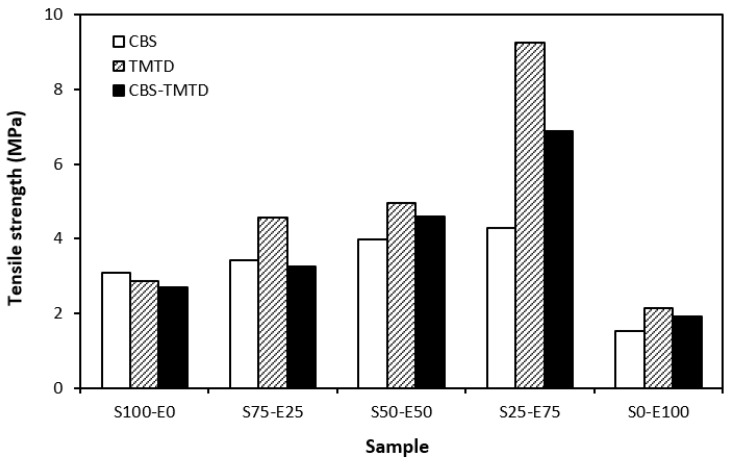
Tensile strength of vulcanizates cured with sulfur systems.

The physical–mechanical properties of sulfur-cured vulcanizates are depicted in Figure 6, Figure 7, Figure 8 and Figure 9. The elongation at the break of vulcanizates correlates, to certain extent, with the cross-link density. As the rubber compounds cured in the presence of CBS (S100–E0, S75–E25, S50–E50) exhibited the lowest cross-link density, those vulcanizates were found to have the highest elongation at break (Figure 6). Also, as the cross-link density of vulcanizates cured in the presence of TMTD and the CBS-TMTD combination showed a decreasing trend with an increasing ratio of EPDM up to the composition of S25–E75, the elongation at break increased in the same direction. The lowest elongation at break was exhibited by vulcanizates based on EPDM with the highest degree of cross-linking. The increasing degree of cross-linking leads to the restriction of the elasticity and mobility of rubber chains, and as a result of which, the elongation at break decreases.

The lowest cross-link density of vulcanizates cured in the presence of CBS was reflected in their lowest modulus (Figure 7). With the application of TMTD and the CBS-TMTD combination, the cross-link density increased, which resulted in an increase in the modulus. In Figure 8, it is shown that the lowest hardness was exhibited by the vulcanizates based on SBR (S100–E0), followed by the vulcanizates based on EPDM (S0–E100). The hardness of materials based on rubber combinations (S75–E25, S50–E50, S25–E75) was slightly higher. The higher cross-link density of vulcanizates cured in the presence of TMTD and the CBS-TMTD combination was responsible for the higher hardness of equivalent vulcanizates. The lowest tensile strength was exhibited by vulcanizates based on EPDM (S0–E100) with no significant influence on the type of accelerator used. The tensile strength of vulcanizates with a designation of S100–E0 was higher. Again, no apparent influence of the accelerator composition on tensile strength was recorded. As shown in Figure 9, in the case of vulcanizates based on rubbers combinations, the tensile strength showed an increasing trend with an increasing ratio of EPDM. Considering the type of accelerator, the highest contribution to the tensile strength was recorded for TMTD. The highest tensile strength was manifested by the vulcanizates with compositions of 25 phr SBR and 75 phr EPDM. The tensile strength of the sample S25–E75 cured with TMTD reached more than 9 MPa, which represents a more than threefold or fourfold increase in tensile strength in comparison with equivalent vulcanizates based on SBR or EPDM, respectively. The highest tensile strength of vulcanizates cured with TMTD can be attributed to the change in the sulfidic cross-links formed within the rubber matrices. As TMTD acts as a sulfur donor, it can be deduced that more disulfidic and polysulfidic cross-links are generated. In general, the vulcanizates with dominant polysulfidic linkages are characterized by higher tensile behavior when compared to vulcanizates in which the chain segments are linked with monosulfidic and disulfidic cross-links. Longer and more flexible polysulfidic cross-links enable higher mobility and elasticity of rubber chains, which contributes to better redistribution of the deformation strains within the rubber matrix [29]. This corresponds with better deformation behavior and higher tensile characteristics of vulcanizates.

### 3.2. Vulcanization Process and Physical–Mechanical Properties of Peroxide-Cured Rubber Compounds

**Figure 10:**
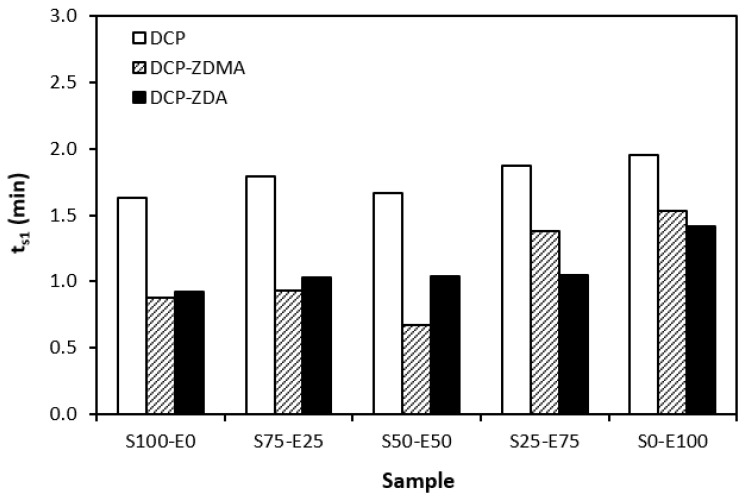
Scorch time *t_s_*_1_ rubber blends cured with peroxide systems.

**Figure 11:**
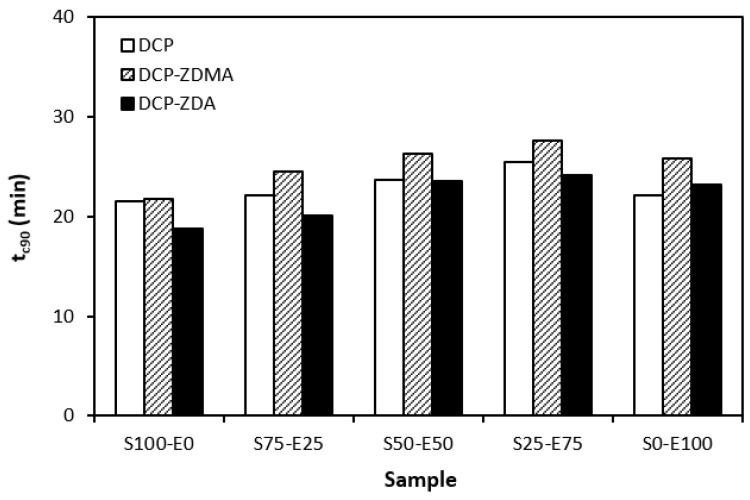
Optimum cure time *t_c_*_90_ of rubber blends cured with peroxide systems.

The influence of peroxide curing composition on the optimum vulcanization time *t_c_*_90_ and scorch time *t_s_*_1_ of rubber compounds is graphically illustrated in Figure 10 and Figure 11. As shown in Figure 10, the highest scorch time was exhibited by the formulations cured with DCP. The application of a co-agent resulted in a decrease in *t_s_*_1_ values. Although, the decrease was only about half a minute, so it can be considered to be not significant. When comparing the scorch time of the blends cured with sulfur systems (Figure 1) and peroxide systems (Figure 10), it becomes apparent that the *t_s_*_1_ of rubber blends cured with peroxide systems is much shorter. The peroxide curing process of rubber blends is a relatively simple process, during which the organic peroxide first decomposes quickly at a curing temperature with the formation of peroxide radicals. The formed radicals then immediately react with rubber chains to form cross-links. This corresponds to a very short scorch time. On the other hand, vulcanization with sulfur is an intricate process, generally running in several stages. The reaction between the accelerators and activators leads to the formation of salt, which subsequently reacts with sulfur to form sulfur-rich transition complexes. This occurs in the induction period, and the length of this period is significantly influenced by the type of accelerator and the sulfur to accelerator ratio.

In Figure 11, it can be seen that there is no significant difference in the optimum cure time depending on the peroxide curing composition. Although, the longest time needed for optimum cross-linking was exhibited by materials cured with DCP and ZDMA. As also shown, the optimum cure time showed a slight increasing tendency with an increasing ratio of EPDM in rubber combinations.

**Figure 12:**
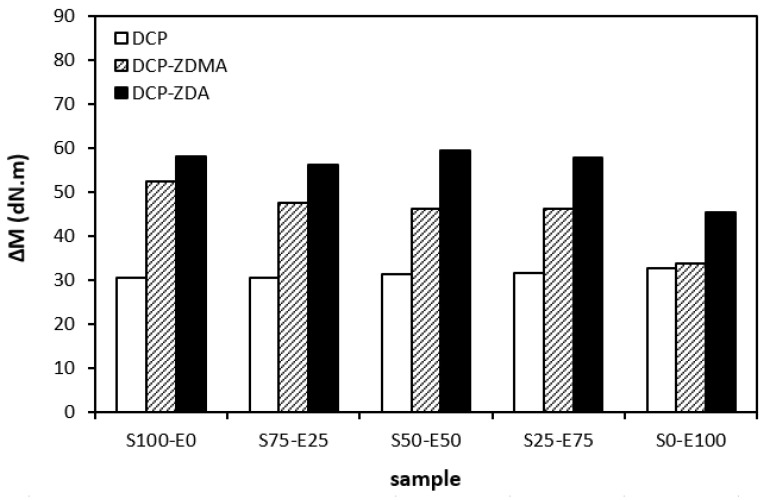
The difference between the maximum and minimum torque Δ*M* of rubber blends cured with peroxide systems.

**Figure 13:**
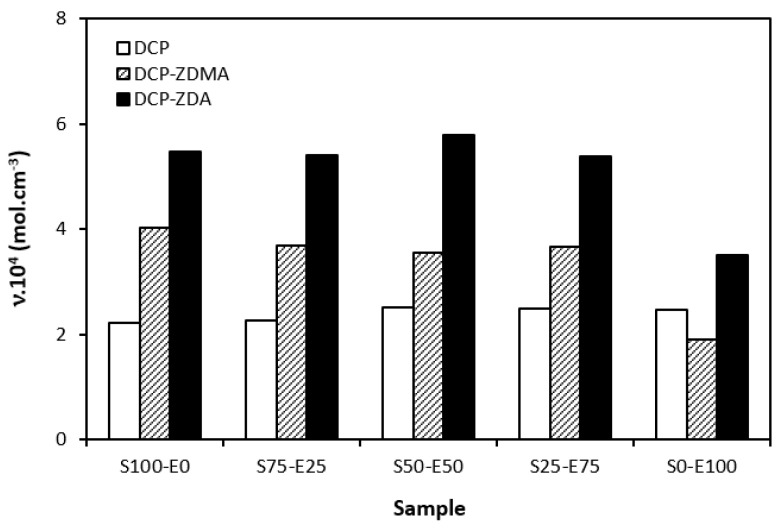
Cross-link density *ν* of vulcanizates cured with peroxide systems.

When comparing the difference between the maximum and minimum torque ∆*M* (Figure 12) and the cross-link density (Figure 13), one can see very similar dependences on the curing system composition and the type of rubber or rubber combinations. It again points to a close relationship between both of those characteristics. The lowest ∆*M* values and cross-link density were demonstrated by materials cured with DCP. As seen, there was almost no difference in the ∆*M* values or the cross-link density depending on the type of rubber of rubber combination. The application of co-agents resulted in an increase in the cross-link density, while a more significant increase was recorded for vulcanizates cured with DCP and ZDA. Co-agents contribute to the increase in peroxide curing efficiency by forming multifunctional cross-links within the rubber matrix. Several reaction mechanisms have been suggested for rubber compounds cured with peroxide in the presence of co-agents. Overall, co-agents can link the rubber chains by forming chemical cross-links between the chain segments; they can also homopolymerize to form interpenetrating networks with rubber chains or high-modulus filler-like domains [30,31,32]. In addition, ion pairs in ZDMA and ZDA can form ion clusters via static electronic interactions. These clusters serve as ionic or physical cross-links with the ability to reduce the stress on the external deformation of the sample, which contributes to the improved physical–mechanical properties [33,34]. The achieved results suggest that both co-agents engage in cross-link network formation within the rubber matrix. A more significant impact on cross-link density was recorded for zinc diacrylate. Thus, it can be deduced that the dominant reaction mechanism of ZDA is the coupling of co-agent molecules onto rubber chains. On the other hand, lower cross-link density of rubber compounds cured with DCP and ZDMA suggests that the dominant reaction pathway for ZDMA is homopolymerization due to the presence of side methyl groups, which can sterically hinder the coupling of co-agent molecules onto rubber chains.

**Figure 14:**
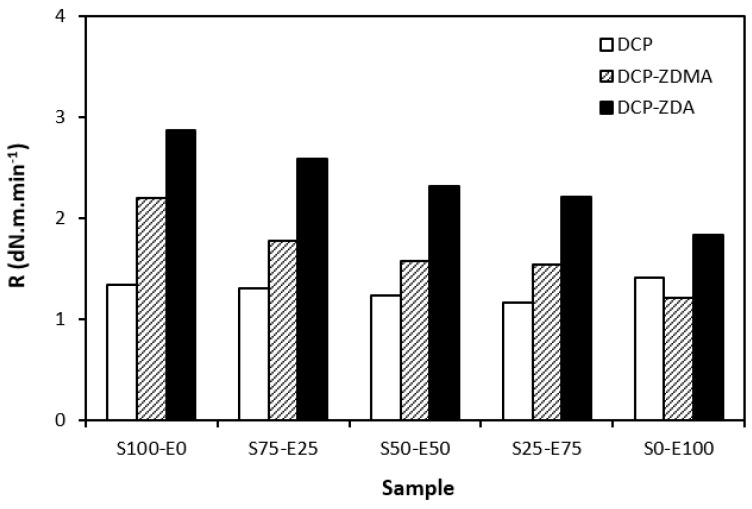
Curing rate *R* of rubber blends cured with peroxide systems.

As shown in Figure 14, the curing rate was the lowest for rubber blends cured with DCP and was found to be not dependent on the rubber compositions. The curing rate of the rubber blends with ZDA and ZDMA was higher and showed a decreasing trend with an increasing amount of EPDM in rubber combinations. It again suggests that the curing process with peroxide systems proceeds faster for SBR.

**Figure 15:**
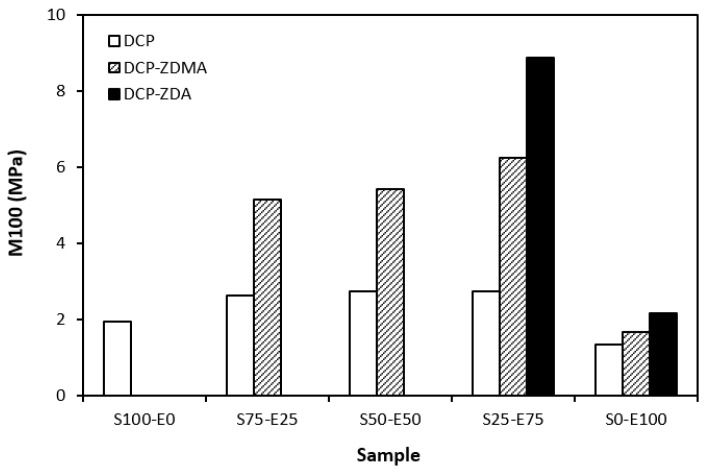
Modulus M100 of vulcanizates cured with peroxide systems.

**Figure 16:**
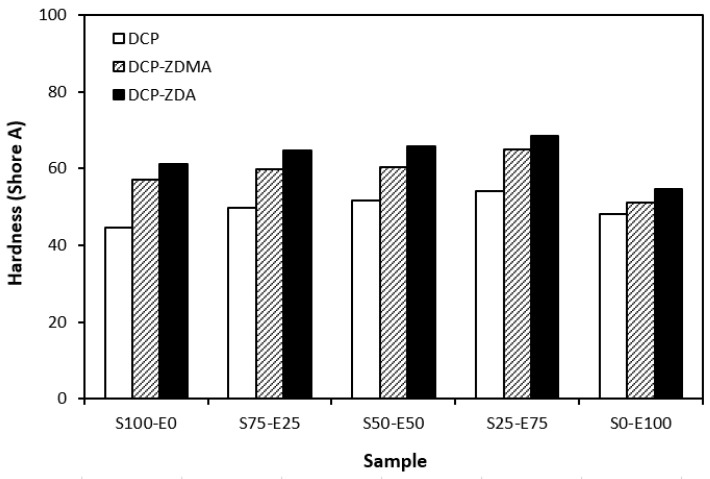
Hardness of vulcanizates cured with peroxide systems.

**Figure 17:**
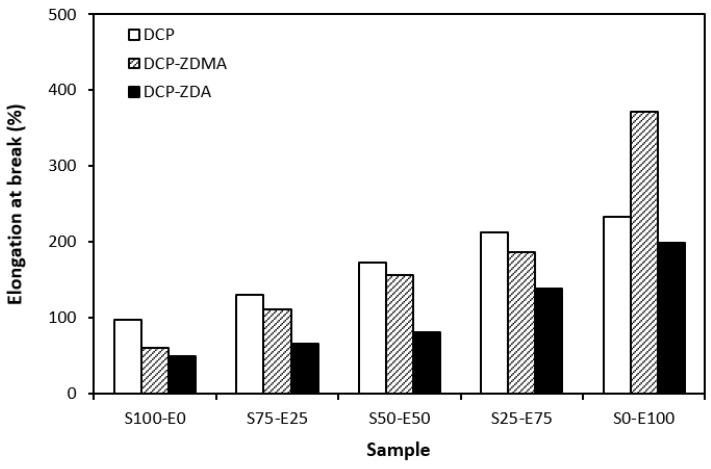
Elongation at break of vulcanizates cured with peroxide systems.

From the graphical dependence of modulus M100 on the rubbers and the curing system composition, one can see that the lowest modulus was exhibited by vulcanizates cured with DCP (Figure 15). This is a consequence of their lowest cross-link density. The application of co-agents resulted in an increase in the cross-link density, which was reflected in the increase in modulus. It was not possible to determine the modulus for several samples, mainly those cured with DCP and ZDA, as due to the high cross-link density, they did not reach 100% elongation. It also becomes interesting that M100 of vulcanizates based on rubber combinations cured with DCP and co-agents was significantly higher when compared to equivalent vulcanizates based on EPDM (S0–E100). The dependences of hardness also followed the trend of cross-link density, suggesting that the highest cross-link density of vulcanizates cured with DCP and ZDA resulted in the highest hardness of the equivalent vulcanizates (Figure 16). The lowest modulus and hardness were found to have vulcanizates based on EPDM (S0–E100) with the lowest cross-linking degree. The elongation at break of vulcanizates showed an increasing trend with an increasing ratio of EPDM in rubber combinations (Figure 17). The highest cross-link density of vulcanizates cured with DCP and ZDA caused the highest restriction of rubber chains mobility, leading to the lowest elongation at break. The highest elongation at break was manifested by vulcanizates based on EPDM (S0–E100) with the lowest cross-link density.

**Figure 18:**
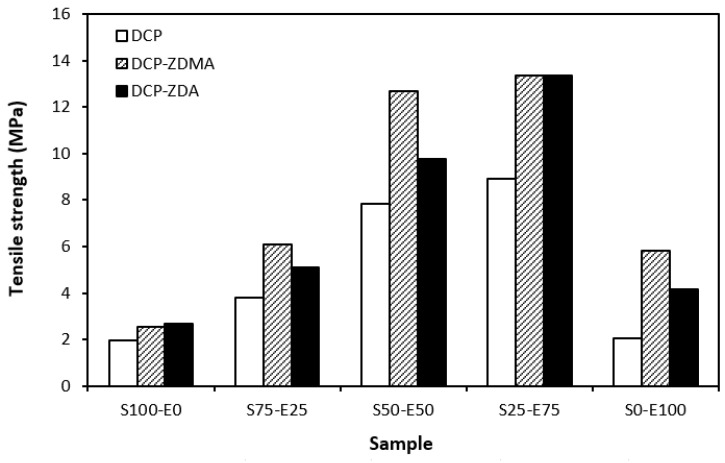
Tensile strength of vulcanizates cured with peroxide systems.

The type and structure of rubber as well as the structure of the formed cross-links are key factors influencing the tensile strength of unfilled vulcanizates. From Figure 18 it becomes apparent that the lowest tensile strength exhibited vulcanizates based on reference SBR and EPDM rubbers (S100–E0 and S0–E100) cured with DCP. While there was almost no influence of co-agents on the tensile strength of the vulcanizate S100–E0, the application of ZDA and ZDMA caused an enhancement in the tensile behavior of the EPDM-based vulcanizate (S0–E100). The tensile strength of vulcanizates based on rubber combinations (S75–E25, S50–E50, S25–E75) was higher and, as in the case of vulcanizates cured with a sulfur system, showed an increasing trend with an increasing ratio of EPDM. When combining SBR and EPDM in 25 phr to 75 phr, the tensile strength of DCP-cured vulcanizate achieved almost 9 MPa, which was more than 7 MPa higher than that of the vulcanizates S100–E0 and S0–E100 (both vulcanizates exhibited a tensile strength equivalent to only 2 MPa). The utilization of co-agents contributed to the formation of more complex cross-linked structures within the rubber matrices through the formation of multifunctional cross-links, which contributed to improvements in the tensile behavior of vulcanizates. Again, the highest tensile strength (remarkable 13 MPa) was manifested by the vulcanizates S25–E75 cured with DCP and both co-agent types.

When comparing the tensile strength of sulfur-cured vulcanizates (including all accelerator systems) and vulcanizates cured with DCP, it is shown that higher tensile strength was exhibited by vulcanizates cured with a sulfur system. This is in line with general knowledge demonstrating that sulfur-cured vulcanizates are characterized by higher tensile behavior compared to peroxide-cured vulcanizates. This can be attributed to the structure of the formed cross-links. Longer and more flexible sulfidic cross-links make rubber chain segments more flexible and elastic. Higher elasticity and mobility of rubber chains leads to better, more uniform redistribution of applied deformation strains onto the rubber matrix, which can bear more strain load without having a negative impact on tensile behavior. On the other hand, short and rigid carbon–carbon bonds restrict the mobility and elasticity of rubber chains. Highly cross-linked rubber sites can act as stress concentrators upon applied deformation strains, which can more easily lead to cracks growing and their propagation. This corresponds to the lower tensile strength of peroxide-cured vulcanizates. Although, with the application of both co-agent types, the tensile strength increased and overcame the tensile strength of sulfur-cured vulcanizates. So, it can be stated that with the proper selection of co-agents, it is possible to fabricate peroxide-cured materials with applicable physical–mechanical properties.

It can also be stated that for both sulfur- and peroxide-cured vulcanizates, the materials based on rubber combinations exhibited higher tensile strength when compared to the equivalent vulcanizates based on SBR or EPDM. The highest tensile strength was demonstrated by vulcanizates with compositions of 25 phr SBR and 75 phr EPDM. This is a very positive aspect, suggesting that with the combination of rubbers, not only can the advantages of both rubbers be combined, but improved tensile behavior can also be achieved.

### 3.3. Dynamical–Mechanical Analysis

Dynamic–mechanical analysis of vulcanizates was performed to investigate the influence of rubber combinations and curing system composition on the visco-elastic properties of vulcanizates. For this purpose, vulcanizates cured with a sulfur system in the presence of TMTD and vulcanizates cured with a combination of DCP and ZDMA were chosen. Those vulcanizates were selected due to their highest tensile characteristics.

**Figure 19:**
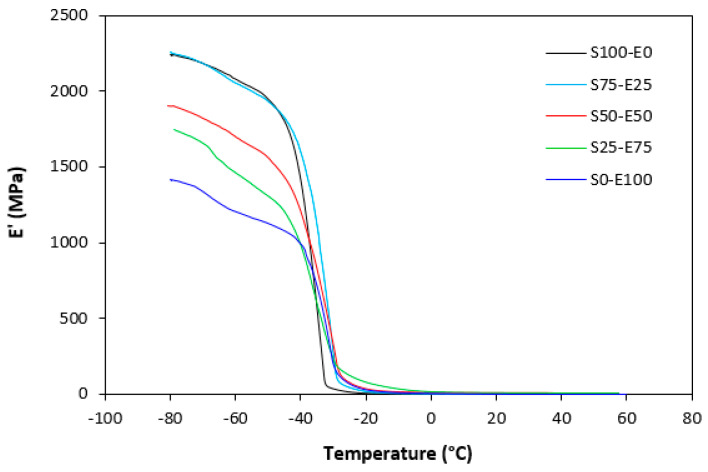
Temperature dependences of storage modulus *E′* for vulcanizates cured with sulfur system.

**Figure 20:**
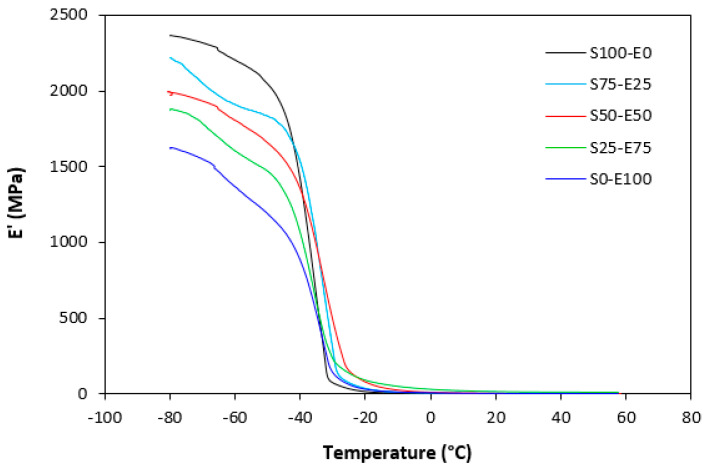
Temperature dependences of storage modulus *E′* for vulcanizates cured with peroxide system.

**Figure 21:**
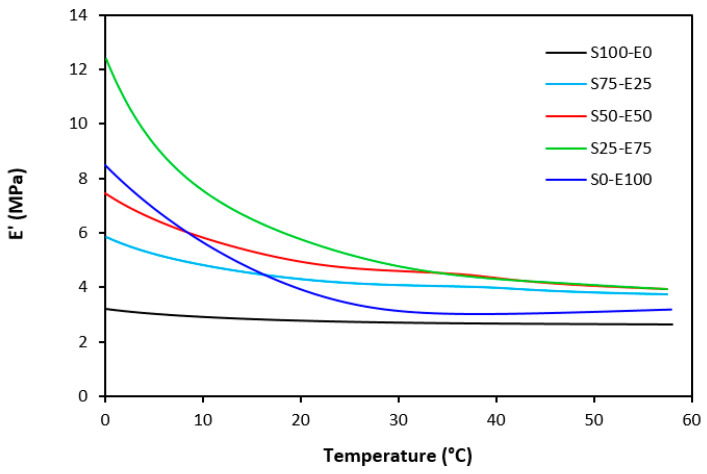
Temperature dependences of storage modulus *E′* for vulcanizates cured with sulfur system (from 0 °C).

**Figure 22:**
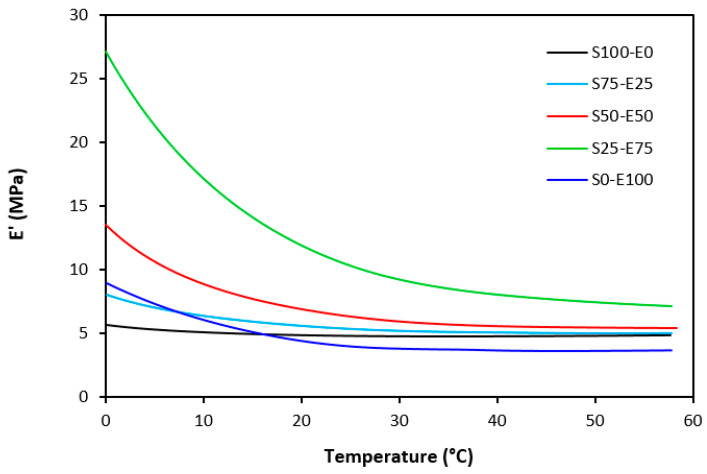
Temperature dependences of storage modulus *E′* for vulcanizates cured with peroxide system (from 0 °C).

The dynamic storage moduli exhibit notable variations in both magnitude and characteristics due to the interplay of intermolecular and intramolecular interactions within the polymer system. Figure 19 illustrates the impact of a blend ratio on the storage modulus (E′) of sulfur-cured vulcanizates. At lower temperatures below the glass transition temperature (Tg), pure SBR (S100–E0) demonstrates the highest E′, while pure EPDM (S0–E100) exhibits the lowest E′, reflecting the distinct structural attributes of each polymer. SBR is a copolymer of styrene and butadiene, which results in a more densely packed polymer structure compared to the terpolymer structure of EPDM, which includes ethylene, propylene, and diene monomers. The more compact structure of SBR generally leads to higher stiffness and a higher storage modulus. A similar trend is observed in Figure 20, where a peroxide curing system was employed, albeit with generally higher modulus values in the glassy region. This phenomenon is ascribed to the greater rigidity conferred by covalent C-C crosslinks compared to more flexible polysulfidic crosslinks. All samples display single transitions, indicative of phase miscibility, yet the curves of EPDM-containing samples feature a less pronounced slope, potentially attributable to the presence of crystalline regions. Upon surpassing the glass transition temperature (Tg), the rubber undergoes a transition from a glassy to a rubbery state, precipitating a decrease in their storage moduli. Logarithmic representation for temperature dependence of storage moduli, depicted in Figure 21 and Figure 22, becomes imperative due to the subtle differences in previous figures. It becomes apparent from these figures that irrespective of the vulcanization system used, the lowest storage moduli in the rubbery region pertain to pure SBR. This phenomenon in the rubbery region can be ascribed to the macromolecules’ ability to impede intramolecular slippage. Despite the expected trend of E′ lying between the values of pure SBR and EPDM, as evident in Figure 21 and Figure 22, this is not the case. Interactions between SBR and EPDM, including intermolecular bonding, may culminate in a more homogeneous blend, thereby yielding a stiffer sample with elevated E′ values in the rubbery state.

**Figure 23:**
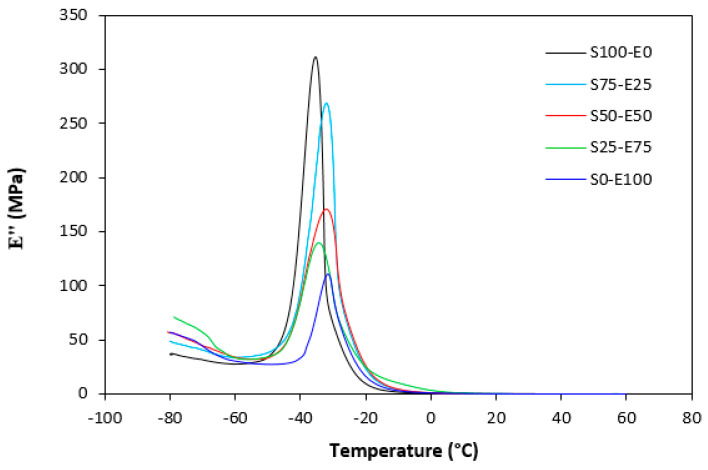
Temperature dependences of loss modulus E″ for vulcanizates cured with sulfur system.

**Figure 24:**
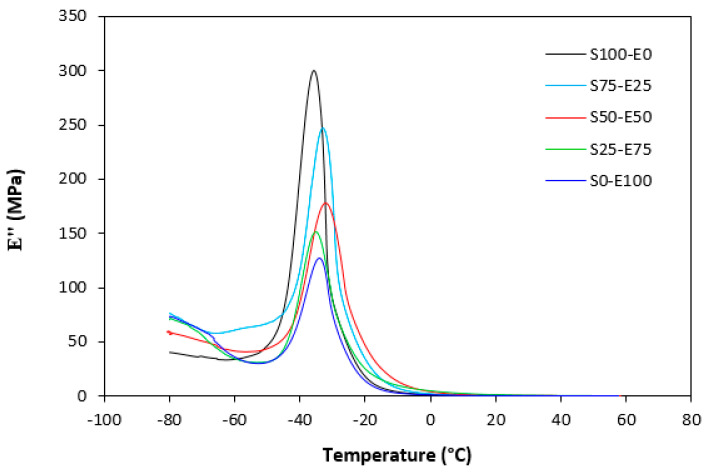
Temperature dependences of loss modulus *E*″ for vulcanizates cured with peroxide system.

**Figure 25:**
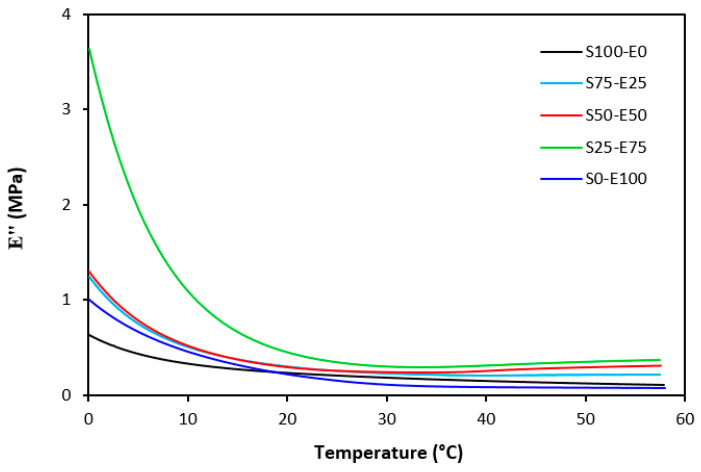
Temperature dependences of loss modulus *E*″ for vulcanizates cured with sulfur system (from 0 °C).

**Figure 26:**
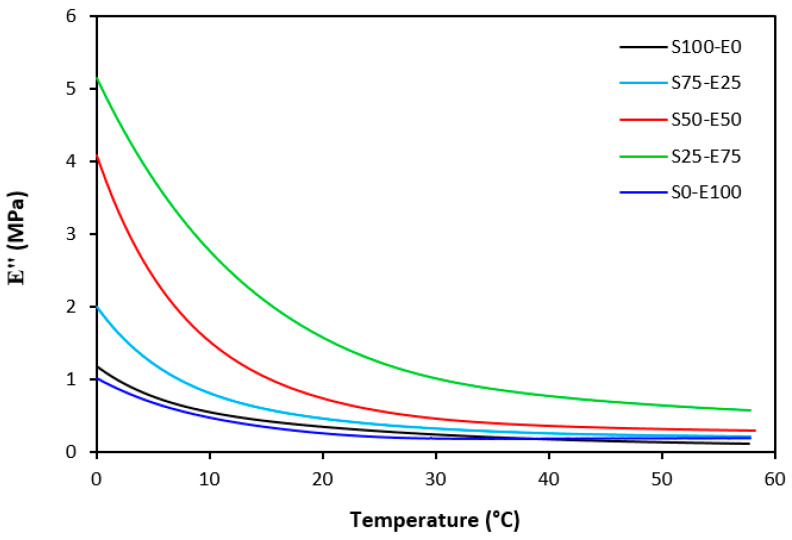
Temperature dependences of loss modulus E″ for vulcanizates cured with peroxide system (from 0 °C).

The loss modulus E″ quantifies the energy dissipation per cycle of deformation during mechanical testing, providing crucial insights into the viscoelastic behavior of materials. In both Figure 23 and Figure 24, singular glass-transition peaks are evident, indicating the presence of a homogeneous phase within the studied polymer blends. Notably, the peaks of S75–E25 blends are marginally shifted to higher temperatures across both curing systems employed. This phenomenon may stem from EPDM being dispersed within a continuous SBR phase, potentially impeding molecular motion and necessitating increased energy to overcome molecular interactions. The area under the peak of the loss modulus curve of pure SBR is observed to be the highest across both vulcanization systems utilized. This observation suggests that SBR exhibits greater viscoelasticity compared to EPDM, enabling it to absorb more energy during deformation. Above the glass transition temperature, the loss modulus reflects heightened molecular mobility, culminating in increased energy dissipation during deformation relative to the glassy state. As depicted in Figure 25 and Figure 26, peroxide vulcanization appears to induce limited chain mobility, resulting in a higher loss modulus at elevated temperatures compared to sulfur vulcanization.

**Figure 27:**
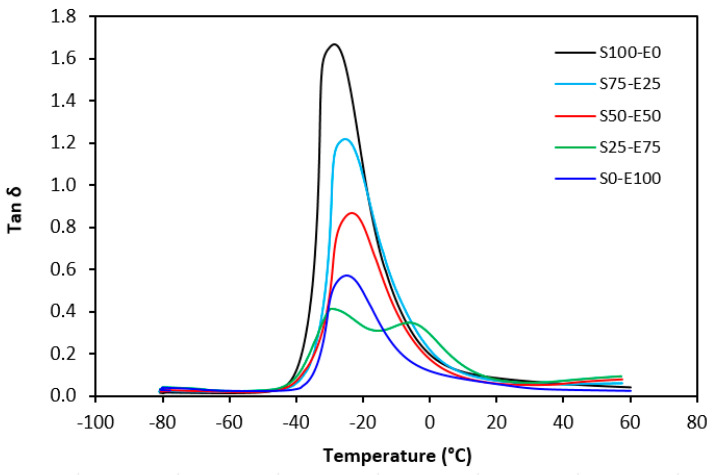
Temperature dependences of loss factor tan δ for vulcanizates cured with sulfur system.

**Figure 28:**
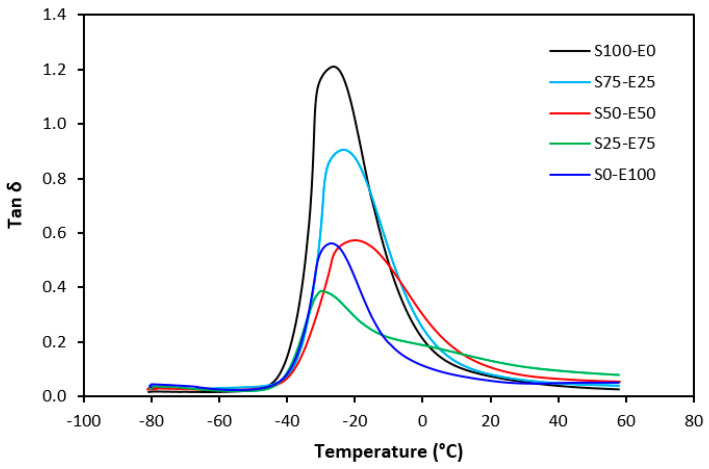
Temperature dependences of loss factor tan δ for vulcanizates cured with peroxide system.

Tan δ serves as the ratio of the loss modulus (E″) to the storage modulus (E′), commonly employed to measure energy dissipation within a material. With a rising temperature, damping peaks within the transition region before decreasing within the rubbery region. Below Tg, damping remains minimal as chain segments are immobilized, yielding primarily elastic deformations with limited molecular motion conducive to viscous flow. Above Tg, damping likewise diminishes as molecular segments gain freedom of movement, encountering minimal resistance to flow. The transition region witnesses heightened damping due to the inception of micro-Brownian motion among macromolecules and their ensuing stress relaxation, although not all segments concurrently partake in such relaxation. The elevated area under the peak of tan δ denotes augmented energy dissipation, similar to E″ elaborated earlier. The temperature dependencies of the loss factor tan δ for both vulcanizate types are delineated in Figure 27 and Figure 28. Peak maxima in tan δ temperature dependencies correspond to the glass transition temperature (Tg) of vulcanizates, with summarized values provided in Table 5. Notably, virgin SBR manifests lower Tg than EPDM. The cross-linking of rubber matrices elevates the glass transition temperature relative to virgin rubbers. Specifically, vulcanizates based on SBR cured with a sulfur system (S100–E0) exhibit lower Tg than their EPDM-based counterparts (S0–E100). Conversely, vulcanizates designated as S100–E0 and S0–E100, cured with a peroxide system, demonstrate practically identical Tg values. This can be attributed to the nearly twofold higher cross-link density of SBR-based vulcanizates compared to those based on EPDM, whereby increased cross-link density restricts rubber chain mobility and elasticity, thus elevating Tg. For both vulcanizate types, those based on an equivalent ratio of both rubbers (S50–E50) exhibit the highest Tg, while those designated as S25–E75 showcase the lowest and nearly identical Tg values (around −29 °C). Figure 27 reveals that the sulfur-cured vulcanizate S25–E75 exhibits two peaks on loss factor temperature dependencies, with the second peak occurring at −6 °C. Given that the occurrence of two transition peaks is typical for block copolymers, it may be inferred that the composition of both rubbers at this ratio precipitates the formation of some block copolymers. Moreover, Figure 28 illustrates that tan δ is higher above Tg, suggesting that the peroxide vulcanization system engenders cross-links between SBR and EPDM that impede segment mobility even above Tg.

## 4. Conclusions

In this work, rubber blends based on SBR, EPDM, and their mutual combinations were cured with sulfur as well as peroxide vulcanization systems.

When considering sulfur curing systems, the longest scorch time as well as the optimum cure time were exhibited by rubber formulations cured with CBS. On the other hand, the highest cure rate and cross-link density was demonstrated by vulcanizates cured with a CBS-TMTD combination. The modulus, hardness, and elongation at break were to a certain extent dependent on cross-link density, suggesting that higher cross-link density of vulcanizates cured with TMTD and the CBS-TMTD combination was reflected in higher modulus and hardness and lower elongation at break of the equivalent vulcanizates. The tensile strength of vulcanizates based on rubber combinations was higher when compared to vulcanizates based on pure SBR and EPDM. The application of TMTD contributed to the highest increase in tensile strength, arguably due to the change in the structure of the formed cross-links. The highest tensile strength was exhibited by the vulcanizate with a composition of 25 phr SBR and 75 phr of EPDM, reaching more than 9 MPa. Similarly, peroxide-cured vulcanizates based on rubber combinations reached a higher tensile strength when compared to vulcanizates based on SBR and EPDM. The application of co-agents resulted in an increase in tensile strength due to the formation of multifunctional cross-links within the rubber matrices. The application of co-agents led to the increase in the modulus and hardness of vulcanizates due to the increase in their cross-linking degree. A high cross-link density was responsible for lower elongation at break of the vulcanizates cured with DCP and co-agents. No significant changes in curing characteristics were recorded depending on the peroxide curing system composition.

## Figures and Tables

**Table 1:** Composition and designation of sulfur-cured rubber blends in the presence of CBS or TMTD (the amount of additives is in phr).

	S100–E0	S75–E25	S50–E50	S25–E75	S0–E100
SBR	100	75	50	25	0
EPDM	0	25	50	75	100
ZnO	3	3	3	3	3
Stearic acid	2	2	2	2	2
Sulfur	1.5	1.5	1.5	1.5	1.5
CBS or TMTD	1.5	1.5	1.5	1.5	1.5

**Table 2:** Composition and designation of sulfur-cured rubber blends in the presence of a combination CBS with TMTD (the amount of additives is in phr).

	S100–E0	S75–E25	S50–E50	S25–E75	S0–E100
SBR	100	75	50	25	0
EPDM	0	25	50	75	100
ZnO	3	3	3	3	3
Stearic acid	2	2	2	2	2
Sulfur	1.5	1.5	1.5	1.5	1.5
CBS	1	1	1	1	1
TMTD	1	1	1	1	1

**Table 3:** Composition and designation of rubber blends cured with DCP (the amount of additives is in phr).

	S100–E0	S75–E25	S50–E50	S25–E75	S0–E100
SBR	100	75	50	25	0
EPDM	0	25	50	75	100
DCP	1	1	1	1	1

**Table 4:** Composition and designation of rubber blends cured with DCP in combination with ZDA or ZDMA (the amount of additives is in phr).

	S100–E0	S75–E25	S50–E50	S25–E75	S0–E100
SBR	100	75	50	25	0
EPDM	0	25	50	75	100
DCP	1	1	1	1	1
ZDA or ZDMA	5	5	5	5	5

**Table 5:** Glass transition temperature Tg and cross-link density *ν* of vulcanizates cured with sulfur and peroxide systems.

	S100–E0	S75–E25	S50–E50	S25–E75	S0–E100
Tg of virgin rubbers (°C)	−34.7				−29.6
Tg of vulcanizates with sulfur system (°C)	−28.5	−25.4	−23.3	−29.2 (−6.2)	−24.9
Tg of vulcanizates with peroxide system (°C)	−26.4	−23.5	−19.8	−29.9	−27
Cross-link density of vulcanizates with sulfur system *ν*·10^4^ (mol·cm^−3^)	1.59	1.47	1.20	0.98	2.95
Cross-link density of vulcanizates with peroxide system *ν*·10^4^ (mol·cm^−3^)	4.02	3.68	3.56	3.66	1.90

## Data Availability

The original contributions presented in the study are included in the article, further inquiries can be directed to the corresponding author.
